# Differential DNA methylation in recovery from shift work disorder

**DOI:** 10.1038/s41598-021-82627-0

**Published:** 2021-02-03

**Authors:** Alexandra Lahtinen, Antti Häkkinen, Sampsa Puttonen, Päivi Vanttola, Katriina Viitasalo, Tarja Porkka-Heiskanen, Mikko Härmä, Tiina Paunio

**Affiliations:** 1 Department of Psychiatry and SleepWell Research Program, Faculty of Medicine, University of Helsinki and Helsinki University Central Hospital, Biomedicum 1, Haartmaninkatu 8, 00290 Helsinki, Finland; 2 Genomics and Biobank UnitDepartment of Public Health Solutions, Finnish Institute for Health and Welfare (THL), PO Box 30, 00271 Helsinki, Finland; 3 Research Program in Systems Oncology, Faculty of Medicine, University of Helsinki, Helsinki, Finland; 4 Work Ability and Working Careersareers, Finnish Institute of Occupational Health, PO Box 40, 00032 Helsinki, Finland; 5 Finnair Health Services, HEL-IF/67, 01053 Finnair, Finland

**Keywords:** DNA methylation, Sleep

## Abstract

The human DNA methylome is responsive to our environment, but its dynamics remain underexplored. We investigated the temporal changes to DNA methylation (DNAme) in relation to recovery from a shift work disorder (SWD) by performing a paired epigenome-wide analysis in an occupational cohort of 32 shift workers (25 men, age = 43.8 ± 8.8 years, 21 SWD cases). We found that the effect of vacation on DNAme was more prominent in the SWD-group as compared to controls, with respect to the amount of significantly differentially methylated positions (DMPs; *P*_*unadj*_ < 0.05) 6.5 vs 3.7%, respectively. The vast majority (78%) of these DMPs were hypomethylated in SWD but not in controls (27%) during the work period. The Gene Ontology Cellular component “NMDA glutamate receptor” (*P*_*FDR*_ < 0.05) was identified in a pathway analysis of the top 30 genes in SWD. In-depth pathway analyses revealed that the Reactome pathway “CREB phosphorylation through the activation of CaMKII” might underlie the recovery. Furthermore, three DMPs from this pathway, corresponding to *GRIN2C*, *CREB1*, and *CAMK2B,* correlated with the degree of recovery (*P*_*unadj*_ < 0.05). Our findings provide evidence for the dynamic nature of DNAme in relation to the recovery process from a circadian disorder, with biological relevance of the emerging pathways.

## Introduction

DNA methylation (DNAme) plays a crucial role during embryonic development. Accordingly, comparisons of various cell types showed that approximately 15–21% of genomic CpGs undergo dynamic changes in the context of normal development^1,2^. Once the DNAme pattern is established, it is maintained during cell divisions in order to retain cell identity^3^. Outside of the early stages of development and within a non-pathological context, DNAme has been considered stable. Data emerging on aging has, however, indicated that DNAme is altered throughout the lifetime, resulting in site-specific hyper- or hypomethylation^3,4^. Despite advances in studying DNAme in developmental contexts as well as across the human lifespan, we still have limited information on the short-term changes in DNAme patterns in humans.

Environmental influences constitute one of the most prominent causes in DNAme changes^5–7^. A study of epigenetic differences in a cohort of monozygotic twins revealed that the patterns of DNAme and histone modifications diverge with aging^8^. Such differences in genetically identical individuals could be explained, to a large extent, by disparate lifestyles, e.g. diet, exercise habits, or smoking. Furthermore, these differences are likely to play an important role in the discordant frequency and onset of many diseases in monozygotic twins^9–11^. All these studies point towards the intriguing hypothesis that DNAme is an important interface between the environment and the genome, and there is a need for specific studies focusing on exploring its dynamic nature. As most epigenome-wide association studies (EWAS) are cross-sectional, the dynamics of DNAme remain poorly explored.

Outside of oncology and studies focused on aging, longitudinal studies in humans are scarce and, to our best knowledge, there are no studies of DNAme on sleep disorders with longitudinal data. Earlier, we reported the results of a cross-sectional genome-wide analysis of DNAme in two cohorts—a community-based sample and an occupational cohort of shift workers^12^. In general, sleep disorders, tiredness, and an increased risk of occupational injuries are associated with working in shifts^13,14^. In the longer perspective, shift work is known to increase the risk for many adverse health effects, such as coronary heart disease (CHD)^15–18^, type 2 diabetes^19–21^, metabolic syndrome^19^, breast cancer^22^, and gastrointestinal disorders, such as peptic ulcer^23^. As a common medical condition in shift workers, SWD is a circadian rhythm sleep–wake disorder characterized by symptoms of insomnia and/or excessive sleepiness associated with working periods^24^. After a vacation period, these symptoms ameliorate, and normal sleep–wake function should be restored. As many as one-third of shift workers develop SWD^25–27^, thus making it a common condition that negatively affects work performance and disrupts family and social life^28–30^. Importantly, the vulnerability to experience SWD symptoms varies remarkably between individuals: some shift workers tolerate and recover from shift work better than others^31–33^, which is at least partially likely to result from genetic influences^34^. Despite a growing interest to study the biology of tolerance and adaptation to shift work, little is known about the molecular processes that underlie recovery from shift work among those with SWD.

In order to explore the dynamic nature of DNAme and to investigate biological processes underlying recovery from SWD, we conducted enrichment analyses of the top findings of the methylation changes for the individuals suffering from SWD. We performed a paired epigenome-wide analysis of DNAme and, for the first time, evaluated the significance of a vacation-specific effect for each CpG in the Illumina 450 K array, separately for the SWD group and the controls. In post-hoc analyses, we assessed the association between the changes in DNAme and the degree of recovery and reported SWD-specific DNA methylation changes that may regulate the restoration of a healthy state.

## Results

### Methylome-wide analyses of the paired Airline data

Our study focused on 32 shift workers (21 cases with SWD, 11 controls, 7 women, age = 43.8 ± 8.8 years) with DNA samples collected during a working period and after a vacation. Based on the estimated change in recovery symptoms for each shift worker, we divided SWD individuals into three groups: well-recovered, recovered, and poorly recovered (See Table 2 in the Supplementary Methods for the characteristics of the SWD participants in the groups).

**Figure 1:**
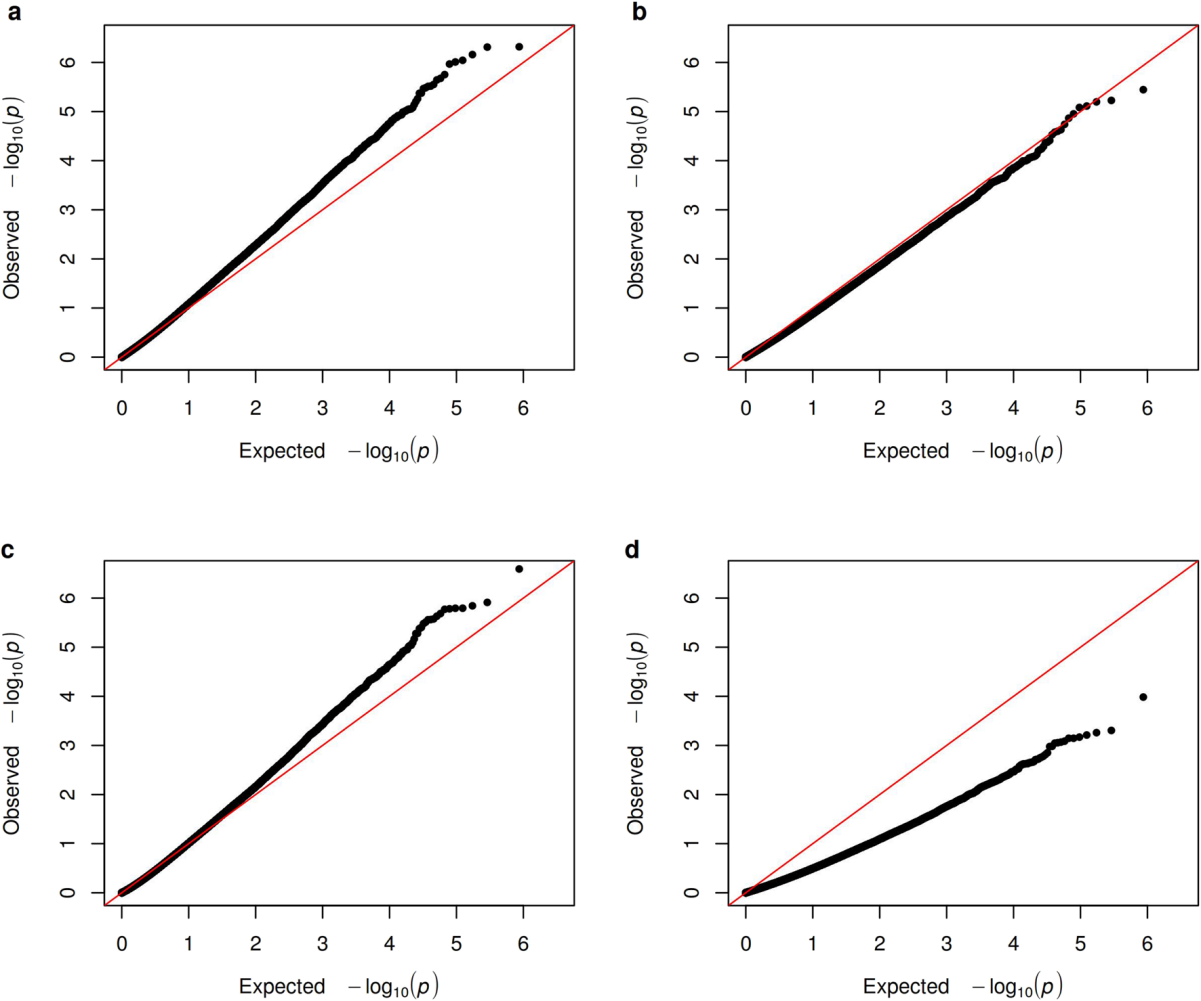
Quantile–quantile plots for (**a**) SWD group, (**b**) control group, (**c**) either group, and (**d**) both groups. Dots represents individual CpG sites and the line identity.

To estimate the effects of being on vacation, as opposed to working on DNAme, we performed EWAS employing a linear regression model (see “Methods” and Supplementary Methods). The effect of vacation was stronger in the SWD group as compared to controls. In SWD, 6.5% (28,419/433,479) of all CpGs were DMPs using an uncorrected *P* < 0.05 (469 CpGs with an FDR-corrected *P* < 0.3). In controls, 3.7% (16,056/433,479) of the CpGs were DMPs using an uncorrected *P* < 0.05 and none of the CpGs remained significant after the multiple testing correction procedure. The resulting quantile–quantile (QQ) plots for all models are shown in the Fig. 1. For the full list presenting the annotated 42,456 DMPs that were differentially methylated in SWD or controls, see Supplementary Data.

We identified a strong effect of vacation on the direction of methylation in the SWD group: 67% (290,430/433,479) of CpGs were hypomethylated during work as compared with vacation, constituting 78% (22,166/28,419) of the DMPs. The corresponding numbers for controls were 40% and 27%, respectively.

We next used the same linear regression model to estimate the effect of vacation in the three recovery groups of SWD individuals separately. Of the DMPs, only 397, 336, and 6,090 (less than 1.5%) were significant in the poorly recovered, recovered, and well-recovered SWD individuals, respectively. However, at 83% (331/397), 88% (299/336), and 79% (4,836/6,090) for the three groups, respectively, the DMPs were hypomethylated during work, as compared with vacation, similar to the overall trend of the SWD group.

Considering the prominent effect of vacation on methylation in the SWD group, we focused on these findings in analysis of biological relevance: gene ontology and exploration of the vacation-sensitive pathways. For the subsequent analysis, we opted to pool the SWD individuals, due to a limited power to analyze sub-groups separately and due to increased hyper- vs hypomethylation as a result of vacation. We selected the top 30 genes (see Supplementary Table S1) ranked by significance (unadjusted *P* value < 1E-5, FDR-corrected *P* value ≤ 0.16) and subjected this set of genes to gene set enrichment analyses.

### Gene ontology enrichment of the vacation-sensitive gene set

#### Gene ontology analyses

To identify the affected biological pathways, we explored three gene set ontology (GO) libraries in Enrichr^35,36^: GO Biological Process, GO Molecular Function, and GO Cellular Component 2018. The top finding from the GO Cellular Component 2018, “NMDA selective glutamate receptor complex” (GO:0017146), was found to be statistically significant at P < 0.05 even after correction for multiple testing (*P* = 8 × 10^–5^ , Benjamini-Hochberg (BH) -corrected *P* = 0.03). The top terms for GO Molecular Function 2018 and GO Biological processes 2018 were “NMDA glutamate receptor activity” (GO:0004972, *P* = 4 × 10^–5^, BH-corrected *P* = 0.05) and “Excitatory chemical synaptic transmission” (GO:0098976, *P* = 4 × 10^–5^, BH-corrected *P* = 0.23), respectively. The findings from GO enrichment analyses are summarized in Supplementary Table S2. Thus, the analyses revealed enrichment of biological processes related to the NMDA glutamate receptor complex in all three ontology libraries.

### In-depth analysis of the vacation-sensitive pathways with reactome

We further explored the pathways involving the vacation-sensitive DMPs by using Reactome. The hierarchical structure of Reactome allows the dissection of the signals into detailed molecular processes^37^. Analysis of the top 30 genes using Reactome 2016 resulted in similar findings as those with GO, adding further evidence for the involvement of changes in glutamate transmission in relation to recovery from SWD (Supplementary Table S3 and Table 1). Furthermore, the results indicated that six out of the ten top findings (*P* value < 0.01) are hierarchically organized under the same parent term “Activation of NMDA receptor upon glutamate binding and postsynaptic events”. This parent pathway is also included in the top-ranked pathway, “CREB phosphorylation through the activation of CaMKII” (Supplementary Fig. S1).

**Table 1:** Enrichment and hypomethylation scores for the significantly differentially methylated CpG sites (DMPs) in the top ten ranked Reactome 2016 pathways.

Rank	REACTOME 2016 pathway	*P* value^a^	Included in the parent pathway^b^	CpGs, N^c^	Genes, N^d^	DMP enrichment score, %		DMP hypo score, %	
						Cases	Controls	Cases	Controls
1	CREB phosphorylation through the activation of CaMKII_Homo sapiens_R-HSA-442729	2.2E-4	Yes	458	15	**8.3**	4.1	73.7	26.3
2	Ras activation upon Ca2 + infux through NMDA receptor_Homo sapiens_R-HSA-442982	2.9E-4	Yes	542	17	7.2	3.7	76.9	40.0
3	Unblocking of NMDA receptor. glutamate binding and activation_Homo sapiens_R-HSA-438066	2.9E-4	Yes	480	17	7.1	3.8	76.5	22.2
4	SALM protein interactions at the synapse_Homo sapiens_R-HSA-8849932	4.9E-4	No	592	20	5.6	3.0	75.8	22.2
5	CREB phosphorylation through the activation of Ras_Homo sapiens_R-HSA-442742	7.5E-4	Yes	913	27	**7.6**	3.2	79.7	34.5
6	Post NMDA receptor activation events_Homo sapiens_R-HSA-438064	1.2E-3	Yes	1078	35	**7.5**	3.4	80.2	35.1
7	Activation of NMDA receptor upon glutamate binding and postsynaptic events_Homo sapiens_R-HSA-442755	1.6E-3	Yes^b^	1136	39	7.3	3.4	80.7	33.3
8	Signaling by Retinoic Acid_Homo sapiens_R-HSA-5362517	1.8E-3	No	727	39	5.6	3.3	78.0	33.3
9	Muscarinic acetylcholine receptors_Homo sapiens_R-HSA-390648	7.5E-3	No	100	5	4.0	2.0	100.0	100.0
10	Synthesis of Dolichyl-phosphate_Homo sapiens_R-HSA-446199	8.9E-6	No	73	6	5.5	2.7	100.0	50.0

^a^*P* value is unadjusted *P* value estimated by Enrichr from analysis of Top 30 genes.

^b^Parent Pathway: “Activation of NMDA receptor upon glutamate binding and postsynaptic events_Homo sapiens_R-HSA-442755”.

^c^Number of all 450 K Illumina CpGs corresponding to the genes enlisted for the pathway.

^d^Number of genes enlisted for this pathway in Reactome 2016 library.

**Figure 2:**
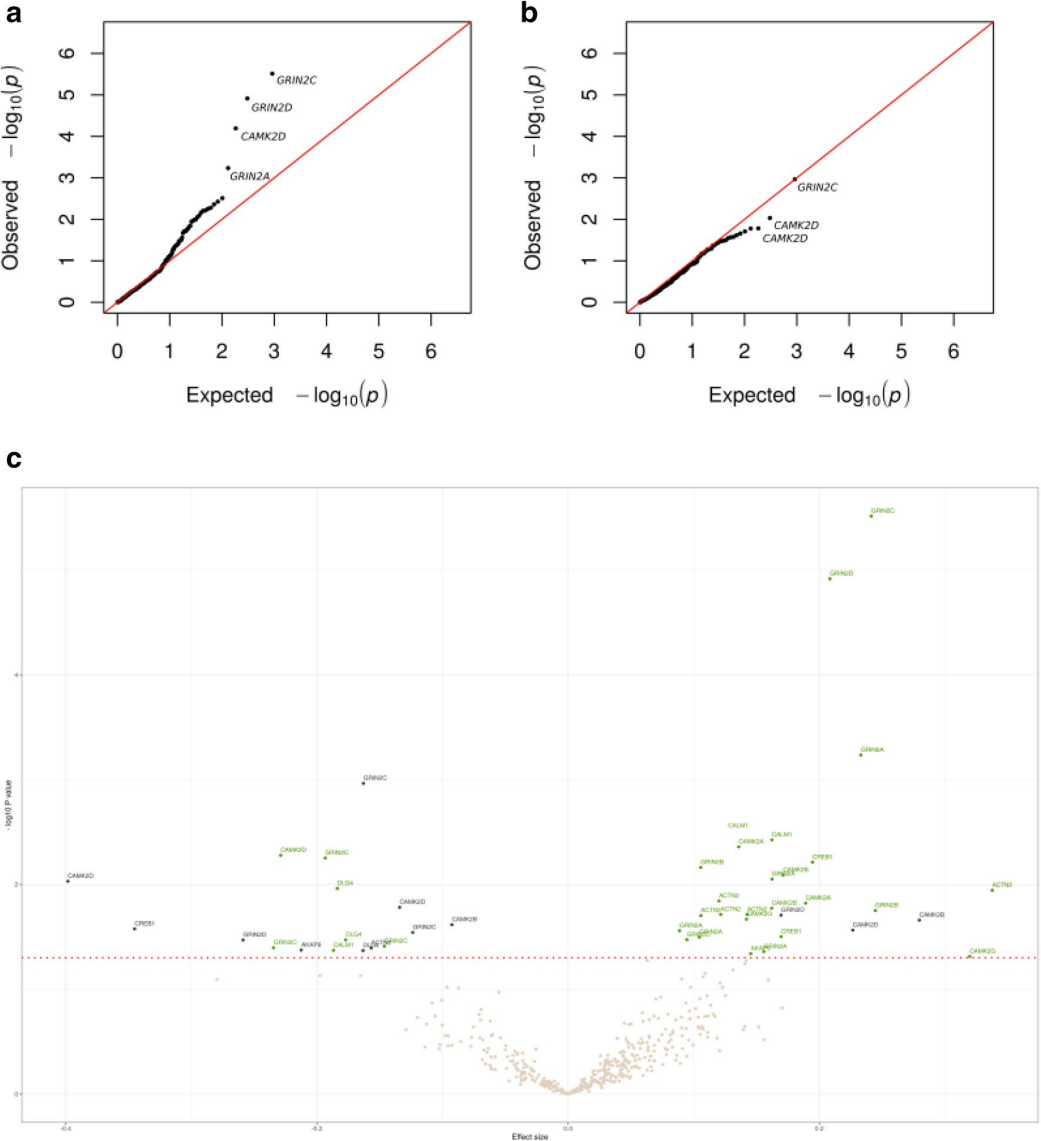
Quantile–quantile plots for the epigenome-wide association in the 458 CpGs corresponding to 15 genes from the pathway “CREB phosphorylation through the activation of CaMKII”: (**a**) SWD group, (**b**) controls. Dots represent individual CpG sites and the lines identity. (**c**) Volcano plot for the Reactome 2016 pathway “CREB phosphorylation through the activation of CaMKII”. Colors of points correspond to: grey, CpGs with unadjusted *P* values > 0.05; green, DMPs for SWD group; black, DMPs for the controls.

For the in-depth pathway analyses, we included CpGs corresponding to all genes listed in each of the Reactome top 10 pathways (genes: n = 5–39 per pathway and CpGs: n = 73–1136 per gene, see Table 1). To investigate the DNAme changes from the paired EWAS study (work vs. vacation), we calculated the DMP enrichment and hypomethylation scores in the CpGs from each pathway both in the SWD and the control groups (Table 1). In the SWD group, we found the highest enrichment score (8.3%, 38/458) for the pathway “CREB phosphorylation through the activation of CaMKII” (CREB-CaMKII pathway). This pathway also showed hypomethylation in 74% of the DMPs (28/38) (for details, see Supplementary Table S4). In controls, the enrichment and hypomethylation scores for this pathway numbered 4.1% (19/458) and 26.3% (5/19), respectively. The QQ plots for CpGs from this pathway are displayed in Fig. 2a (SWD group) and 2b (controls), which show that the significant DMPs are frequent in cases but not in controls. Thus, our results provide evidence for a relatively robust involvement of CpGs from genes of the CREB-CaMKII pathway specific to the SWD group.

In order to visualize the magnitude, direction, and significance of the DNAme changes induced by vacation for this pathway, we utilized a scatter plot (‘volcano plot’) visualization (Fig. 2c). The largest changes in hypermethylation, along with the highest statistical significance in SWD group were observed for the DMPs corresponding to the genes encoding NMDA receptor subunits GRIN2C and GRIN2D. We also discovered DMPs corresponding to 13/15 genes (*GRIN2A*, *GRIN2B*, *GRIN2C*, *GRIN2D*, *CAMK2A*, *CAMK2B*, *CAMK2D*, *CAMK2G*, *CREB1*, *CALM1*, *DLG4*, *ACTN2*, *AKAP9*) in this pathway in the SWD group, suggesting that virtually all genes in this pathway had one or more CpGs affected by the vacation.

### In-depth analysis of DMPs from the CREB-CamKII pathway

#### Correlations of vacation M-values with the recovery in SWD group

**Figure 3:**
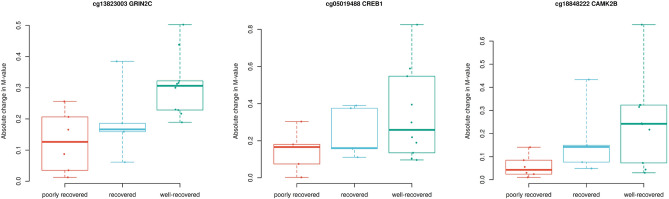
Boxplots of the three discovered differentially methylated CpG in the Reactome 2016 pathway “CREB phosphorylation through the activation of CaMKII” for the groups with different degree of recovery. Y-axis represents the absolute change in the M-values from work to vacation. Colors of the dots correspond to the different groups: red, poorly recovered; blue, recovered; green, well-recovered. The horizontal line is median, box is 25 to 75%, the whiskers denote data, and the dots represent individual samples.

We further investigated whether the changes in the methylation values (M-values) of the 38 DMPs from the CREB-CaMKII pathway correlate with the degree of recovery in the SWD group based on data from on-site questionnaires. We found 3/38 DMPs to correlate at *P* < 0.05 with the degree of recovery, none of which remained significant after multiple testing correction (Table 2 and Supplementary Table S5). The change in M-values correlated inversely with the degree of recovery for cg13823003 (*GRIN2C*), cg05019488 (*CREB1*), and cg18848222 (*CAMK2B*), as all three sites showed hypomethylation during working period as compared with vacation. Figure 3 illustrates the biggest absolute change in the M-values of individuals with the highest degree of recovery.

**Table 2:** Correlation between the methylation changes with the changes in recovery symptoms: the three identified DMPs and the corresponding genes.

DMP	Gene	Chromosome	Coordinate	Spearman correlation, r	*P* value	q value
cg13823003	*GRIN2C*	17	70367644	− 0.622	0.003	0.205
cg05019488	*CREB1*	2	208173979	− 0.493	0.023	0.488
cg18848222	*CAMK2B*	7	44225262	− 0.485	0.026	0.488

^a^*P* value, uncorrected *P* value, 2-tailed; q value, FDR-corrected *P* value.

#### SWD vs. controls during work

We then compared the methylation levels of the 38 DMPs in the SWD group and the controls during the working period. In the SWD group, 28/38 of the sites were hypomethylated and, respectively, 10/38 were hypermethylated, during the working period, as compared with vacation. The majority of the hypomethylated (20/28) sites showed lower M-values in the SWD group as compared with the controls; accordingly, the majority (9/10) of the hypermethylated showed higher M-values in SWD (one site, cg13823003 from *GRIN2C*, with a nominally significant difference at *P* < 0.05, analysis of variance (ANOVA) (Supplementary Table S6).

The results of the in-depth analysis of the CREB-CaMKII pathway are summarized in the Table 3. Of all the DMPs identified, the findings with cg13823003, corresponding to gene *GRIN2C,* were the most robust and exhibited all the following properties: a nominally significant *P* value < 0.05 in the secondary analyses of recovery; ANOVA analysis indicated an altered behavior during the working period; and the site was among the 30 most significant sites for the pathway analysis.

**Table 3:** Summary of the results of each analysis for the 38 DMPs from the pathway “CREB phosphorylation through the activation of CaMKII”.

Gene	DMP	EWAS cases		ANOVA at work *P* value	Secondary analyses of recovery	
		Sign at work	*P* value		Spearman r	*P* value
ACTN2	cg11197458	Hypo	0.020	0.677	− 0.270	0.237
ACTN2	cg12297935	Hypo	0.014	0.408	− 0.181	0.432
ACTN2	cg23109559	Hypo	0.011	0.499	0.265	0.246
ACTN2	cg23233975	Hypo	0.019	0.355	− 0.133	0.566
ACTN2	cg26406150	Hypo	0.019	0.914	0.050	0.831
AKAP9	cg16688376	Hypo	0.046	0.822	− 0.083	0.720
CALM1	cg01311654	Hyper	0.042	0.355	0.334	0.138
CALM1	cg04712435	Hypo	0.003	0.834	0.345	0.125
CALM1	cg05077358	Hypo	0.004	0.650	0.408	0.066
CAMK2A	cg03873049	Hypo	0.004	0.592	0.015	0.949
CAMK2A	cg06620397	Hypo	0.015	0.233	− 0.318	0.160
CAMK2B	cg18848222	Hypo	0.017	0.602	− 0.485	0.026
CAMK2B	cg23997477	Hypo	0.008	0.132	0.149	0.519
CAMK2D	cg13801347	Hypo	0.010	0.915	0.008	0.972
CAMK2D	cg17237111	Hyper	6.399E−05	0.236	0.024	0.917
CAMK2D	cg20391984	Hyper	0.005	0.379	0.077	0.742
CAMK2G	cg02032166	Hypo	0.049	0.921	− 0.169	0.463
CAMK2G	cg08797625	Hyper	0.010	0.939	− 0.087	0.707
CAMK2G	cg17422824	Hypo	0.021	0.499	0.102	0.660
CREB1	cg05019488	Hypo	0.006	0.765	− 0.493	0.023
CREB1	cg14129040	Hypo	0.031	0.290	− 0.197	0.391
CREB1	cg15749141	Hyper	0.006	0.342	0.353	0.116
DLG4	cg13729891	Hyper	0.034	0.170	− 0.179	0.439
DLG4	cg21218476	Hyper	0.011	0.482	− 0.114	0.622
GRIN2A	cg00534626	Hypo	0.001	0.099	− 0.032	0.890
GRIN2A	cg01344243	Hypo	0.044	0.204	0.068	0.768
GRIN2A	cg06829391	Hypo	0.009	0.414	0.136	0.558
GRIN2A	cg09461286	Hypo	0.028	0.219	0.191	0.408
GRIN2A	cg16378117	Hypo	0.032	0.942	0.257	0.262
GRIN2B	cg03777288	Hypo	0.042	0.073	− 0.085	0.715
GRIN2B	cg14351692	Hypo	0.007	0.694	0.185	0.421
GRIN2B	cg23942984	Hypo	0.018	0.162	− 0.083	0.720
GRIN2C	cg09722397	Hyper	0.006	0.363	0.068	0.768
GRIN2C	cg13823003	Hypo	3.076E−06	0.037	− 0.622	0.003
GRIN2C	cg18035537	Hyper	0.040	0.147	− 0.188	0.414
GRIN2C	cg21997766	Hyper	0.039	0.248	0.121	0.602
GRIN2D	cg08525508	Hypo	0.034	0.078	0.269	0.239
GRIN2D	cg12546181	Hypo	1.217E−05	0.086	0.106	0.647

### Global DNAme patterns across the recovery groups of SWD group

**Figure 4:**
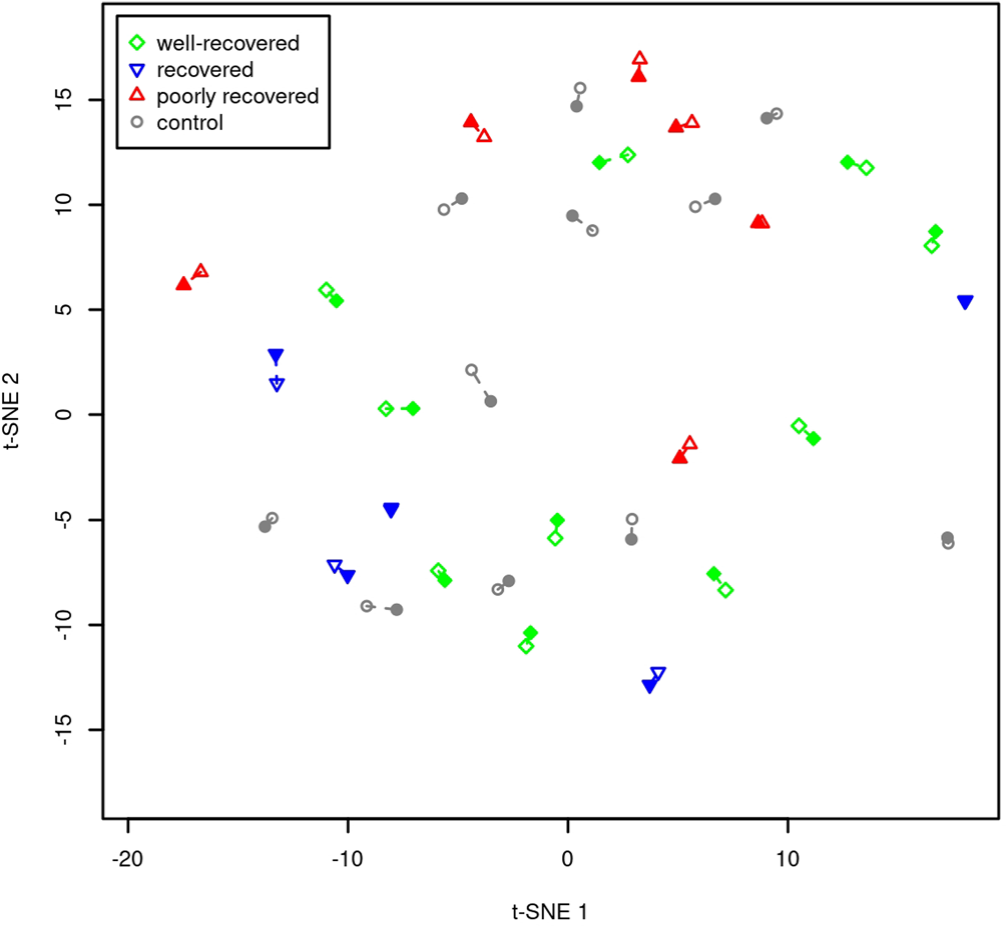
T-SNE mapping of the global DNA methylome profiles across the recovery groups of SWD group. Colors of the dots correspond to the different groups: red, poorly recovered; blue, recovered; green, well-recovered; grey, controls. Hollow markers represent samples collected during work and filled ones during vacation, dashed lines connecting the samples of each patient.

In order to explore global DNAme patterns across the recovery groups of SWD individuals and to compare them with DNAme patterns of controls, we performed a t-SNE mapping of the global DNA methylome profiles (Fig. 4). Globally the methylation profiles of the well-recovered individuals resembled the profiles of the controls, while the poorly recovered and recovered individuals cluster separately at specific regions of the DNA methylome space.

## Discussion

In order to gain insight into the dynamic nature of DNAme, we explored the effect of vacation on DNAme compared to a working period in shift workers. Our hypothesis was that among the shift workers with SWD, recovery from insufficient sleep would be reflected in the changes of DNAme patterns in white blood cells. This study used paired data from an occupational cohort of shift workers, with and without SWD, for genome-wide analysis of DNAme from whole blood samples, in combination with information from sleep diaries and questionnaires collected during work and after vacation. To the best of our knowledge, this is the first study to longitudinally examine dynamic DNAme changes in relation to a manifesting sleep disorder and recovery from it.

The main finding of this study is that the effect of vacation on DNAme was more prominent in the SWD group, as compared to the controls, with dynamic changes observed in 6.5% of the DMPs. The observed changes included a gain in methylation induced by vacation, which was more remarkable for the individuals suffering from SWD, as compared with the controls. An overall trend towards global DNAme hypomethylation was earlier reported in several epigenome-wide association studies conducted in the cohorts of shift workers^38–40^. Moreover, this finding is in accordance with our previous study^12^, where we observed a loss of methylation in individuals suffering from insufficient sleep. In the current study, we further confirmed that insufficient sleep during work is associated with hypomethylation, while the recovery from SWD symptoms during vacation corresponded to the restoration of methylation.

Gene enrichment analyses of the DMPs which showed the largest changes after vacation in the SWD group revealed pathways related to the activity of a glutamatergic NMDA receptor. These pathways included GRIN-subunits of NMDA receptor, several Ca^2+^/calmodulin-dependent protein kinases (CaM kinases), and the transcription factor CREB1. This study’s focus on longitudinal observations of the shift workers enabled us to narrow down a wide group of pathways to the specific molecular process involving only a few genes. In depth analysis revealed the Reactome pathway “CREB phosphorylation through the activation of CaMKII” to have the highest enrichment of methylome changes. This pathway includes the genes encoding for cAMP responsive element binding protein 1 *(CREB1)*, calcium/calmodulin dependent protein kinase II beta *(CAMK2B)*, and glutamate ionotropic receptor NMDA type, subunit 2C *(GRIN2C)*.

Recovery dynamics in shift workers in the SWD group were found to be highly individual, with some shift workers reporting full recovery, while others still mentioned sleepiness and insomnia even after the vacation. We found that the largest changes in recovery symptoms were accompanied by the largest DNAme changes of three CpGs from the CREB-CaMKII pathway. One of them, a CpG site cg13823003 from *GRIN2C*, was remarkably robust through our study, showing statistical significance both in ANOVA analysis during the working period and in the secondary analyses of recovery. This site was also among the top DMPs obtained from the paired EWAS. According to the Ensembl Regulatory Build in Ensembl (http://www.ensembl.org/info/genome/funcgen/index.html) and Illumina 450 K annotation, cg13823003 locates at the CpG Island in the *GRIN2C* promoter and CTCF-binding site. In the same CpG Island in the TSS200 region, we found two adjacent CpG sites, which were differentially hypermethylated during work in SWD cases (See Supplementary Table S7 for details). Interestingly, one of these sites, cg09722397, was identified earlier in our previous study in a sample of adolescents suffering from depression and comorbid insomnia^41^. In coherence with the present study, where this site was hypermethylated in SWD cases during work, it was hypermethylated also among the adolescent cases, as compared to age and gender-matched healthy controls. However, the opposite direction of methylation observed in these three CpGs located at the promoter of *GRIN2C* (two hyper- and one hypomethylated) presents a challenge for an attempt to propose the possible consequences for gene expression.

The members of the CREB-CAMK pathway that we identified in the present study belong to the Ca^2+^-dependent hyperpolarization pathway, which has been implicated in the regulation of sleep duration^42^. Several other studies exploring knockouts of the genes activating the Ca^2+^-dependent hyperpolarization pathway resulted in the same conclusion: the activity of these genes is associated with the cortical capacity to evoke slow-wave oscillations, which, in turn, is proportional to animal sleep duration^43–45^.

As our study was carried out in the DNA samples from peripheral blood, we explored what is known about possible functions of such key members of CREB-CAMK pathway, as GRIN-subunits of NMDA receptor, CaM kinases, and CREB1 in the white blood cells.

Human lymphocytes express various receptors, including NMDA glutamate receptors. The activation of these receptors may be involved in lymphocyte survival and differentiation of T-cells, and modulations of immune functions, as suggested by studies using NMDA receptor- binding, structural glutamate analogues^46^.

CRE elements have been identified in the promoters of many T-cell genes, indicating that transcription factor CREB might be involved in processes, such as activation and proliferation of T and B cells, as well as monocyte survival^47^. Less is known about calcium-calmodulin pathway, though some rodent studies evidenced involvement of CaM kinases^48^ and calmodulin in the cytokine production, T cell activation and proliferation^49^. Based on our findings from the white blood cells, it is possible to suggest that the vacation-induced changes in the activity of some members of CREB-CAMK pathway might be regulated via DNA methylation. The observed alterations in DNAme of GRIN-subunits, CREB, and CaM kinases may indicate immune function-related differences between individuals with SWD and controls. Several previous studies have shown an association between continuous sleep insufficiency, common among the shift workers, and alterations in host defence and immunity^50–55^.

Our analysis of the global DNAme profiles in the recovery groups of individuals with SWD showed no clear differences in the degree of changes in poor versus well-recovered groups. This supports our findings from epigenome-wide association study and gene enrichment analyses, suggesting that the vacation did not affect DNAme in a genome-wide fashion, but affected the CpGs locally in specific pathways.

There are several limitations in this study. Firstly, the sample size was limited, and larger samples are required to detect statistically robust findings on a larger scale. However, a carefully a priori selected study design with two data collection points allowed us to detect statistically significant differences by applying a statistical model and assessing differences separately for the groups. Furthermore, the SWD and non-SWD status was assessed by both objective and subjective measurements, as in addition to the questionnaires, we performed both actigraphy monitoring and evaluated sleep diaries, as required by the International Classification of Sleep disorders-Third Edition (ICSD-3) criteria^56^. The second limitation is that in the EWAS study of the whole blood samples we did not perform adjustment for the blood cell type composition. However, prior to analyses, we estimated that our groups did not differ in their blood cell type composition, nor did this composition change from working period to vacation in the same individual. Thus, most of the significant changes in DNAme observed after the vacation are unlikely to be explained by the cell type composition. Thirdly, we lacked objective measurements of sleep insufficiency and relied on self-reported data in the post-hoc analyses. Nevertheless, it is known that there are large individual differences in shift work tolerance^31–33^ and self-reported data allowed us to capture such variability among the shift workers and identify specific sites that are sensitive for the degree of recovery. We also lacked the information regarding the chronotype and sleep/wake habits of our subjects. The fourth limitation relates to the statistically significant differences in age observed between SWD cases and controls. Since age has a known impact on the DNAme, we included age as a covariate in our model. The fifth limitation of our study was that we could not assess the functional significance of the identified DNAme changes due to absence of corresponding gene expression data. The last potential limitation is related to the possible order effect of this study: we investigated the changes occurring from the SWD-symptoms towards the recovery, but the changes from recovered to the diseased state could be asymmetric.

Despite these weaknesses, our study is one of the few existing paired EWASes investigating short-term changes in DNAme patterns in humans. We observed prominent methylation changes within several months in the SWD group and depicted a molecular brain-specific pathway associated with these changes. Furthermore, we distinguished specific molecules that might indicate the degree of recovery of a shift worker with SWD. Further studies in larger cohorts are required to fully uncover the mechanisms and validate their health-related potential.

## Conclusion

Our findings suggest that the DNAme of shift workers shows a specific response to vacation, particularly in individuals suffering from SWD symptoms. The observed differences in methylation patterns take place in processes related to NMDA receptor activity. In particular, our analysis identified the CpG sites in *GRIN2C*, *CREB1*, and *CAMK2B* as putatively important indicators of recovery in a shift worker with SWD symptoms. Further, our results demonstrate that even a two-week period of vacation exerts changes in the human DNA methylome – a highly dynamic and complex regulatory mechanism that we are only beginning to understand.

## Methods

### Study design and participants

#### Paired measurements

Whole blood samples, sleep diaries, and questionnaires on SWD symptoms were collected twice in the lab for each participant. The ‘working period’ measurement was performed during the shift worker’s working period: blood samples were collected between 7 a.m. and 11 a.m. from a healthy participant, with no infection episode in the previous 7 days. On the same morning, the participant completed the questionnaire in the lab. ‘Work’ sample collection dates varied between May 2012 and June of 2013. The ‘vacation’ measurement was performed after at least two weeks of vacation (at the last day of vacation). The blood sample was collected between 7 a.m. and 11 a.m., and each participant completed the questionnaire. ‘Vacation’ sample collection dates varied between June 2012 and August 2013.

#### Participants

32 shift workers aged 27–60 years (22% women) from a Finnish airline company were involved^31^. The work schedule during the last 12 months included regular night shifts (a minimum of 3 h of work between 11 p.m. and 6 a.m.) and/or early morning shifts (starting by 6 a.m.).

#### SWD status

The SWD and non-SWD (control) status was assessed by both subjective and objective measurements: (1) shift working day -specific insomnia and sleepiness symptoms reported in the questionnaire, and (2) a working shift-related reduction of the total sleep time reported in the sleep diaries and actigraphy monitoring, as required by the International Classification of Sleep disorders-Third Edition (ICSD-3) criteria^56^. 21 shift workers (24% women, age = 41.0 ± 8.9 years) constituted the SWD group: a participant reported symptoms of insomnia and/or sleepiness “often/continuously” during working period only and had a reduced total sleep time. 11 shift workers (18% women, age = 49.2 ± 9.1 years) lacking the symptoms were deemed as controls. The SWD status assessment is described in detail in our previous studies^12,31^.

#### Recovery groups in the SWD group

To assess the degree of recovery in the SWD group, we used two questions from the on-site questionnaire that measured important aspects of recovery for shift workers: (A) “How often do you not feel fresh after sleep?” and (B) “How often do you feel daytime sleepiness?”. The change in recovery symptoms for each shift worker in the SWD group was estimated from the difference as follows:

$$CHANGE\_SYMPTOMS = \, {\left( {A \, + \, B} \right)_{WORK}}-- \, {\left( {A \, + \, B} \right)_{VACATION}}$$

Tables 1 and 2 in Supplementary Methods summarize the participants’ characteristics.

### Infinium human methylation 450K bead chip methylation measurements

The DNA extraction, CpG methylation, and quality control procedures are described in detail in Lahtinen et al.^12^. DNA was extracted from the whole blood samples using standard methods (See Supplementary Methods for details) and methylation measurements were performed using Infinium HumanMethylation450k BeadChip (Illumina, Inc., San Diego, CA, USA). Methylation data preprocessing and quality control were performed using R package “*minfi*” v1.18.4^57^ in R software v3.6.1 (https://www.r-project.org/). 433,479 probes passed the quality control.

### Data analyses

#### Methylome-wide paired analysis

To estimate an effect of being on vacation as opposed to working on the methylation of each of the *n* probes in the *m* individuals, we employed the following linear regression model:

$${y_{i, \, v\left\{ i \right\}, \, j}} = \, \left( {1 - {h_i}} \right){\beta_{h = 0,j}} + {h_i}{\beta_{h = 1,j}} + \, \left( {1 - {h_i}} \right){v_i}{\beta_{v - w,h = 0,j}} + {h_i}{v_i}{\beta_{v - w,h = 1,j}} + {D_{i,j}}{\beta_{D,j}} + {\varepsilon_{i,v\left\{ i \right\},j}}$$

$$for\,i\,in \, \left\{ {1, \ldots ,m} \right\},{v_i}in \, \left\{ {0,1} \right\},j\,in \, \left\{ {1, \ldots ,n} \right\}$$

where *y*_i, 0, j_ and *y*_i, 1, j_ are the methylation of the *i*:th individual at the *j*:th probe when on work and vacation, respectively; *h *_*i*_ is a flag indicating if the individual belongs to the SWD group or control (*h*_*i*_ = 1 for a control and *h*_*i*_ = 0 for the SWD group); *v*_*i*_ is a flag indicating if the measurement is from a vacation period or not (*v*_*i*_ = 0 for the working period, *v*_*i*_ = 1 for vacation); *D*_*j*_ are the nuisances such as age, sex, plate, alcohol consumption, and smoking status; and *ε*_*i*,1,*j*_ are the errors, assumed to be independent and identically distributed zero-mean normal random variables (i.e. the effects were found using the ordinary least squares procedure). In this model, *β *_*h*=0, *j*_ and *β *_*h*=1, *j*_ represent the group average methylation for the SWD and control groups (at the *j*:th probe) when working, respectively (after removing the nuisance effects); *β *_*v*-*w*, *h*=0, *j*_ and *β *_*v*-*w*, *h*=1, *j*_ represent the effect of vacation (versus work) on the methylation in the two groups; and *β *_*D*, *j*_ the nuisance effects.

The significance of the effect of vacation (versus work) on the methylation of the *j*:th probe, *β *_*v*-*w*, *h*, *j*_, was tested using a variance ratio (F) test. The relevant null hypotheses considered no effect on each, either, or both groups, that is, the null hypothesis *β *_*v*-*w*, *h*=0, *j*_ = 0 and *β *_*v*-*w*, *h*=1, *j*_ = 0 tested for an effect on either group; *β *_*v*-*w*, *h*=0, *j*_ = 0 tested for an effect in the SWD group, while *β *_*v*-*w*, *h*=1, *j*_ = 0 tested for an effect in the control group; and the hypothesis *β *_*v*-*w*, *h*=0, *j*_ = 0 or *β *_*v*-*w*, *h*=1, *j*_ = 0 (which can be evaluated using a composite null model) tested for an effect in both groups. Finally, the acquired *P* values were adjusted to control false discovery rate (FDR) in multiple hypothesis testing using the Benjamini–Hochberg procedure. We defined a CpG site to be hyper- or hypomethylated based on the value of the beta coefficient in our tests, with hypermethylation characterized by a positive value. To estimate the relative proportions of cell type components in the whole blood we used a statistical method described by Houseman et al.^58^. T-test was used to investigate the differences in the cell type compositions between and within the groups.

In order to obtain the gene names associated with the CpGs, we used Infinium HumanMethylation450k BeadChip annotation data (Illumina, Inc., San Diego, CA, USA) and R software v3.6.1 (https://www.r-project.org/).

#### Pathway analyses using Enrichr

Enriched terms were explored using Enrichr (https://amp.pharm.mssm.edu/Enrichr/)^36^. We focused on three GO libraries: GO Biological Process, GO Molecular Function, and GO Cellular Component 2018. A list of genes ranked by *P* values served as the input and *P* < 0.05 (after Benjamini–Hochberg correction) was used to determine significance.

#### In-depth pathway analysis using Reactome 2016

For the top 10 Reactome 2016 pathways ranked by unadjusted P values, we extracted the full gene set from Reactome 2016 library and calculated the (1) enrichment score (%) and (2) hypomethylation score (%), for both the SWD and control groups.

#### Post-hoc analyses of the changes in M-values in the recovery groups

To estimate the change in the methylation values in the SWD group, we calculated the difference between the M-values measured during the working period and vacation, as follows:

$$CHANGE\_METHYLATION \, = \, M - VALU{E_{WORK}}-- \, M - VALU{E_{VACATION}}$$

The Spearman correlation between the M-value changes and the recovery symptom changes (*CHANGE_SYMPTOMS,* as defined earlier) was used to estimate recovery association. A *P* value < 0.05 (after Benjamini–Hochberg correction) was used to determine significance.

#### Post-hoc analyses during working period

ANOVA was performed for the methylation measured during the working period. A *P* value < 0.05 (after Benjamini–Hochberg correction) was used to determine significance.

#### Assessment of global DNAme profiles in the recovery groups

 T-SNE mapping of the global methylome profiles was performed using software Rtsne 0.15^59^. Perplexity was set to 10 (cf. 64 samples), and 10,000 iterations were run.

### Study approval

Sample collection and study design were performed according to the principles of the Declaration of Helsinki and were approved by Coordinating Ethics Committee of the Helsinki and Uusimaa Hospital District. All participants provided written informed consent.

## Supplementary Information

Supplementary Information 1.

Supplementary Information 2.

Supplementary Information 3.

## Data Availability

The ethical approval limits the individual-level data availability from the Airline cohort and prohibits the authors from making the data set publicly available. Data are available from the corresponding author (Tiina Paunio) upon ethical approval from the Coordinating Ethics Committee of the Helsinki and Uusimaa Hospital District.
