# Supplementary material for: Differential DNA methylation in recovery from shift work disorder
Source: Sci Rep. 2021 Feb 3;11:2895. doi: 10.1038/s41598-021-82627-0 (PMC7858604; doi:10.1038/s41598-021-82627-0)
Supplement: Supplementary file 3 — Supplementary Information 3. [file 41598_2021_82627_MOESM3_ESM.doc]

**Supplementary Methods for ”Differential DNA methylation in recovery from shift work disorder”**

| Alexandra Lahtinen^1,2^, Antti Häkkinen^3^, Sampsa Puttonen^4^, Päivi Vanttola^4^, Katriina Viitasalo^5^, Tarja Porkka-Heiskanen^1^, Mikko Härmä^4^, Tiina Paunio^1, 2^ |
| --- |
| **Affiliations:** |
| ^1^SleepWell Research Program, Faculty of Medicine, University of Helsinki, Helsinki, Finland |
| ^2^Genomics and Biobank Unit, Department of Public Health Solutions, Finnish Institute for Health and Welfare (THL), PO Box 30, FI-00271, Helsinki, Finland |
| ^3^Research Program in Systems Oncology, Faculty of Medicine, University of Helsinki, Helsinki, Finland |
| ^4^Work ability and Working careers, Finnish Institute of Occupational Health, PO Box 40, FI-00032, Helsinki, Finland |
| ^5^Finnair Health Services, HEL-IF/67, FI-01053 Finnair, Finland |

**Study design and the participants**

*Paired measurements.* This study comprised whole blood samples, sleep diaries, and questionnaires on shift work disorder (SWD) symptoms collected online and twice in the lab for each participant. The first measurement, ‘working period’, was performed during a working period of a shift worker: we collected the blood sample between 7 a.m. and 11 a.m. from a healthy participant, with no infection episode in the previous 7 days. No participant donated a blood sample after the night shift. In the same morning, the participant filled the questionnaire in the lab. Online questionnaire was filled before the lab visit or later during the field study. ‘Work’ sample collection dates varied between May 2012 and June of 2013. The second measurement, ‘vacation’, was performed after at least two weeks of vacation (on the last day of vacation). We collected the blood sample between 7 a.m. and 11 a.m., and each participant completed the same questionnaire in the lab. ‘Vacation’ sample collection dates varied between June 2012 and August 2013. We did not obtain any details on the vacation of the participants (duration, place, time zone, nor travel mode). The intervals between the samplings varied between 1 and 6 months in the SWD group and between 2 and 6 months in the control group. We found that these did not differ significantly (t-test, *P* = 0.13). Further, we found no difference in the season of the vacation between the groups (chi-squared test, *P* = 0.61). We obtained paired DNA samples and complete data from the questionnaires, including the SWD status, for the 32 shift workers.

*Participants.* Thirty-two shift workers aged 27-60 years (22% women) from a Finnish airline company participated in this study (1). The shift work schedule during the last 12 months had to include regular night shifts (minimum 3 hours of work between 11 p.m. and 6 a.m.) and/or early morning shifts (starting by 6 a.m.). All participants provided the information about alcohol consumption and smoking status.

*SWD status.* The SWD and non-SWD (control) status was assessed by both subjective and objective measurements: (1) shift working day -specific insomnia and sleepiness symptoms reported in the questionnaire, and (2) a working shift-related reduction of the total sleep time reported in the sleep diaries and actigraphy monitoring, as required by the International Classification of Sleep disorders-Third Edition (ICSD-3) criteria (2). Twenty-one shift workers (24% women, age = 41.0 ± 8.9 years) constituted the SWD group: a participant reported symptoms of insomnia and/or sleepiness “often/continuously” during working period only and had a reduced total sleep time. Eleven shift workers (18% women, age = 49.2 ± 9.1 years) lacking sleepiness and insomnia symptoms during work and vacation were deemed as controls. The SWD status assessment is described in detail in our previous studies (1, 3).

*Recovery groups in the SWD group.* All shift workers completed questionnaires in the laboratory in the same morning the blood sample was collected. The questions covered various aspects of symptoms, such as daytime sleepiness, feeling fresh after sleep, and difficulties to fall asleep or stay awake. We also asked the sleep duration per 24 hours on the working day (at working period) and on the day off (both on working period and on vacation), as well as the date when the questionnaire was completed. To assess the degree of recovery in the SWD group, we used two questions from the onsite questionnaire that measured important aspects of recovery of shift workers: (A) “How often do you not feel fresh after sleep?” and (B) “How often do you feel daytime sleepiness?”. The answers to both questions ranged from 1 to 4, meaning (1) “less than once a week”, (2) “1-2 days a week”, (3) “3-5 days a week”, and (4) “often/continuously”. We estimated the change in recovery symptoms for each shift worker in the SWD group by calculating the difference between the score reported during working period and vacation, as follows:

*CHANGE_SYMPTOMS = (A + B)_WORK_ – (A + B)_VACATION_*

The well-recovered group included 10 individuals whose change in symptoms equaled to 2 or 3 units. The recovered group combined 5 individuals whose change in symptoms equaled to 1 unit. The poorly recovered group comprised 6 individuals who demonstrated no change in symptoms (0) or got worse (-1). See Tables 1 and 2 below for the summary of the characteristics of the participants.

Table 1. Characteristics of the 32 shift worker participants.

|  | **All (N=32)** | **SWD (n = 21)** | **Controls (n = 11)** |
| --- | --- | --- | --- |
| Age (SD), years | 43.8 (8.8) | 41.0 (8.9) | 49.2 (9.1) |
| Women | 7 (22%) | 5 (24%) | 2 (18%) |
| Shift work disorder status | 21 (66%) | 21 (100%) | 0 |
| Smoking status |  |  |  |
| *"Never smoked"* | 11 (34%) | 8 (38%) | 3 (27%) |
| *"Quit"* | 18 (56%) | 11 (52%) | 7 (64%) |
| *"Current smoker"* | 3 (9%) | 2(10%) | 1 (9%) |
| Alcohol consumption |  |  |  |
| *"Once a month or rarer"* | 1 (3%) | 0 | 1 (9%) |
| *"2-4 times per month"* | 12 (38%) | 10 (48%) | 2 (18%) |
| *"2-3 times per week"* | 14 (44%) | 8 (38%) | 6 (54%) |
| *"at least 4 times per week"* | 5 (16%) | 3 (14%) | 2 (18%) |
| Blood cell composition at WORK (SD), % |  |  |  |
| *CD8T* | 6.2 (3.8) | 7.0 (3.6) | 4.9 (4.0) |
| *CD4T* | 15.9 (5.6) | 15.4 (5.6) | 17.0 (5.7) |
| *NK* | 8.7 (5.0) | 9.2 (4.9) | 7.6 (5.1) |
| *Bcell* | 5.1 (2.3) | 5.1 (2.0) | 5.1 (3.0) |
| *Mono* | 8.0 (2.8) | 7.9 (2.9) | 8.2 (2.7) |
| *Gran* | 57.7 (8.6) | 57.1 (9.0) | 58.9 (8.1) |
| Blood cell composition at VACATION (SD), % |  |  |  |
| *CD8T* | 6.2 (3.9) | 6.8 (3.2) | 5.1 (4.9) |
| *CD4T* | 15.6 (6.4) | 15.5 (7.4) | 15.7 (4.1) |
| *NK* | 9.1 (5.4) | 9.4 (5.6) | 8.5 (5.4) |
| *Bcell* | 4.9 (2.9) | 5.0 (3.1) | 4.7 (2.6) |
| *Mono* | 8.6 (2.3) | 8.8 (2.1) | 8.2 (2.6) |
| *Gran* | 57.3 (9.8) | 56.2 (9.0) | 59.3 (8.1) |
| Vacation season |  |  |  |
| *“winter”* | 8 (25%) | 6 (29%) | 2 (18%) |
| *“summer”* | 12 (38%) | 8 (38%) | 4 (36%) |
| *“spring”* | 10 (31%) | 5 (24%) | 5 (46%) |
| *“autumn”* | 2 (6%) | 2 (9%) | 0 |
| Recovery groups for the SWD group |  |  |  |
| *Well-recovered* | - | 10 (48%) | - |
| *Recovered* | - | 5 (24%) | - |
| *Poorly recovered* | - | 6 (28%) | - |
| *Data are mean (SD), n (%). Blood cell types are CD8T, CD8 T cells; CD4T, CD4 T cells; NK, natural killers; Bcell, B cells; Mono, monocytes; Gran, granulocytes.* | | | |

Table 2. Characteristics of the SWD participants by their recovery group.

|  | **Well-recovered (n=10)** | **Recovered  (n = 5)** | **Poorly recovered (n = 6)** |
| --- | --- | --- | --- |
| Age (SD), years | 40.8 (6.7) | 39.2 (10.6) | 43.0 (10.3) |
| Women | 3 (30%) | 1 (20%) | 1 (17%) |
| Smoking status |  |  |  |
| *"Never smoked"* | 2 (20%) | 4 (80%) | 2 (33%) |
| *"Quit"* | 6 (60%) | 1 (20%) | 4 (67%) |
| *"Current smoker"* | 2 (20%) | 0 | 0 |
| Alcohol consumption |  |  |  |
| *"Once a month or rarer"* | 0 | 0 | 0 |
| *"2-4 times per month"* | 6 (60%) | 2 (40%) | 2 (33%) |
| *"2-3 times per week"* | 3 (30%) | 2 (40%) | 3 (50%) |
| *"at least 4 times per week"* | 1 (10%) | 1 (20%) | 1 (17%) |
| *Data are mean (SD), n (%)* | | | |

**Infinium HumanMethylation450K BeadChip methylation measurements**

The DNA extraction, CpG methylation, and quality control procedures were described in Lahtinen *et al.* (3). In brief, four millilitres of whole blood was obtained and DNA was extracted using DNA Blood Mini Kits (Qiagen, Germany). DNA was quantified using PicoGreen® (Life Technologies, NY, USA) in a 96-well plate format (samples were normalized to 100 ng/µL) and stored at -70ºC. Bisulphite conversion of 500 ng of each DNA sample was performed using the EZ-96 DNA Methylation-Gold Kit according to the manufacturer’s protocol (Zymo Research, CA, USA). Next, 4 µl of bisulphite-converted DNA was used for the hybridization on the Infinium HumanMethylation450k BeadChip (Illumina, Inc., San Diego, CA, USA), following the Illumina Infinium HD Methylation protocol. Methylation measurements were performed at the core facility of the Institute of Genomics at the University of Tartu. We preprocessed the raw intensity data files and generated M-values, as described in the R package “*minfi*” v1.18.4 (4). We corrected methylation values for background and applied normalization with a subset quantile normalization approach (SWAN) (5). The inspection of the control probe signals revealed no outliers and no sex discrepancies after checking the sex prediction. The quality control steps included the removal of the probes with a low (< 95%) detection rate at *P* value < 0.01 and probes located on sex chromosomes. As described in Chen *et al.* (6), we also excluded known cross-reactive probes and probes containing a SNP. After the quality control steps, 433,479 probes were used for the analyses. Methylation data preprocessing and quality control were performed using R software v3.6.1 (https://www.r-project.org/).

**Data analyses**

*Methylome-wide paired analysis.* To estimate an effect of being on vacation as opposed to working on the methylation of each of the *n* probes in the *m* individuals, we employed the following linear regression model:

*y* _i, v{i}, j_ = (1-*h _i_*) *β _h_*_=0_, *_j_* + *h _i_* *β _h_*_=1_, *_j_* + (1-*h _i_*) *v _i_* *β _v_*-*_w_*, *_h_*_=0_, *_j_* + *h _i_* *v _i_* *β _v_*-*_w_*, *_h_*_=1_, *_j_* + *D _i_*, *_j_* *β _D_*, *_j_* + *ε _i_*, *_v_*{*_i_*}, *_j_*

for *i* in {1, ..., *m*}, *v _i_* in {0,1}, *j* in {1, ..., *n*}

where *y* _i, 0, j_ and *y* _i, 1, j_ are the methylation of the *i*:th individual at the *j*:th probe when on work and vacation, respectively; *h _i_* is a flag indicating if the individual belongs to the SWD group or control (*h _i_*=1 for a control and *h _i_*=0 for the SWD group); *v _i_* is a flag indicating if the measurement is from a vacation period or not (*v _i_*=0 for the working period, *v _i_*=1 for vacation); *D*:, *_j_* are the nuisances such as age, sex, plate, alcohol consumption, and smoking status; and *ε _i_*_, 1_, *_j_* are the errors, assumed to be independent and identically distributed zero-mean normal random variables (i.e. the effects were found using the ordinary least squares procedure). In this model, *β _h_*_=0_, *_j_* and *β _h_*_=1_, *_j_* represent the group average methylation for the SWD and control groups (at the *j*:th probe) when working, respectively (after removing the nuisance effects); *β _v_*-*_w_*, *_h_*_=0_, *_j_* and *β _v_*-*_w_*, *_h_*_=1_, *_j_* represent the effect of vacation (versus work) on the methylation in the two groups; and *β _D_*, *_j_* the nuisance effects.

The significance of the effect of vacation (versus work) on the methylation of the *j*:th probe, *β _v_*-*_w_*, *_h_*, *_j_*, was tested using a variance ratio (F) test. The relevant null hypotheses considered no effect on each, either, or both groups, that is, the null hypothesis *β _v_*-*_w_*, *_h_*_=0_, *_j_*=0 and *β _v_*-*_w_*, *_h_*_=1_, *_j_*=0 tested for an effect on either group; *β _v_*-*_w_*, *_h_*_=0_, *_j_*=0 tested for an effect in the SWD group, while *β _v_*-*_w_*, *_h_*_=1_, *_j_*=0 tested for an effect in the control group; and the hypothesis *β _v_*-*_w_*, *_h_*_=0_, *_j_*=0 or *β _v_*-*_w_*, *_h_*_=1_, *_j_*=0 (which can be evaluated using a composite null model) tested for an effect in both groups. Finally, the acquired *P* values were adjusted to control false discovery rate (FDR) in multiple hypothesis testing using the Benjamini-Hochberg procedure. We defined a CpG site to be hyper- or hypomethylated based on the value of the beta coefficient in our tests, with hypermethylation characterized by a positive value. We used Infinium HumanMethylation450k BeadChip annotation data to obtain the gene names. For the enrichment analyses, we selected the top thirty genes ranked by *P* values corresponding to the CpGs from the SWD group. We used QQ plots to visualize the results of methylome-wise paired analyses. Fig. 1 and 2 were created using R package “*qqman*” v0.1.4. Analyses were conducted using R software v3.6.1 (https://www.r-project.org/).

*Pathway analyses using Enrichr tools.* To identify the enriched terms for the genes corresponding to CpGs identified in the previous step, we used an integrative and freely available web-based platform Enrichr (<https://amp.pharm.mssm.edu/Enrichr/>) (7). We focused on three gene set ontology libraries - GO Biological Process, GO Molecular Function, and GO Cellular Component 2018. A list of genes ranked by *P* values served as the input and *P* < 0.05 (after Benjamini-Hochberg multiple testing correction) was used to determine statistical significance.

*In-depth pathways analysis using Reactome 2016.* We investigated the top ten Reactome 2016 pathways ranked by P values, with unadjusted *P* value < 0.01. For each pathway, we extracted the full gene set from Reactome 2016 library and calculated two parameters for the SWD and control groups separately: (1) enrichment score, % and (2) hypomethylation score, %. The calculations were performed as follows:

1. Number of the EWAS DMPs (CpGs with unadjusted P values < 0.05)/ Number of all CpGs in Illumina 450K array corresponding to all genes listed in the Reactome 2016 library for a given pathway, %
2. Number of the EWAS DMPs that showed hypomethylation at work/ Number of all CpGs in Illumina 450K array corresponding to all genes listed in the Reactome 2016 library for a given pathway, %;

To visualize the changes in methylation values induced by vacation for the pathway of interest, we utilized a scatter plot (‘volcano plot’). Fig. 3 was created using R packages “*ggplot2*” v3.2.1 and “*ggpubr*” v0.2.4 on R software v3.6.1 (https://www.r-project.org/).

*Post-hoc analyses of the changes in M-values in the recovery groups.* In order to estimate the change in the methylation values for each of the 38 DMPs for each shift worker in the SWD group, we calculated the difference between the M-values measured during working period and vacation, as follows:

*CHANGE_METHYLATION = M-VALUE_WORK_ – M-VALUE_VACATION_*

The changes in recovery symptoms for each shift worker in the SWD group were calculated as the difference between the score reported during working period and vacation, as follows:

*CHANGE_SYMPTOMS = (Q5 + Q6)_WORK_ – (Q5 + Q6)_VACATION_*

The correlation between the changes in M-values and the changes in the symptoms was quantified using Spearman correlation. *P* value < 0.05 (after Benjamini-Hochberg multiple testing correction) was used to determine statistical significance of a nonzero correlation. Post-hoc analyses and data visualizations were conducted using R packages “*ggplot2*” v3.2.1 and “*ggpubr*” v0.2.4, R software v3.6.1 (https://www.r-project.org/).

*Post-hoc analyses in during working period.* The analyses of variance (ANOVA) were performed for the methylation data measured at working period. *P* value < 0.05 (after Benjamini-Hochberg multiple testing correction) was used to determine statistical significance. Post-hoc analyses were conducted using R software v3.6.1 (https://www.r-project.org/).

*Assessment of global DNAme profiles in the recovery groups.* T-SNE mapping of the global methylome profiles was performed using software Rtsne v0.15 (8). Perplexity was set to 10 (cf. 64 samples), and 10,000 iterations were run.

**Study approval**

Sample collection and study design were performed according to the principles of the Declaration of Helsinki and were approved by Coordinating Ethics Committee of the Helsinki and Uusimaa Hospital District. All participants provided written informed consent.

**References**

1. Vanttola P, Harma M, Viitasalo K, Hublin C, Virkkala J, Sallinen M, et al (2019): Sleep and alertness in shift work disorder: findings of a field study. *Int Arch Occup Environ Health* 92:523-533.

2. American Academy of Sleep Medicine (2014) International Classification of Sleep Disorders.

3. Lahtinen A, Puttonen S, Vanttola P, Viitasalo K, Sulkava S, Pervjakova N, et al (2019): A distinctive DNA methylation pattern in insufficient sleep. *Sci Rep* 9:1193-018-38009-0.

4. Aryee MJ, Jaffe AE, Corrada-Bravo H, Ladd-Acosta C, Feinberg AP, Hansen KD, et al (2014): Minfi: a flexible and comprehensive Bioconductor package for the analysis of Infinium DNA methylation microarrays. *Bioinformatics* 30:1363-1369.

5. Maksimovic J, Gordon L, Oshlack A (2012): SWAN: Subset-quantile within array normalization for illumina infinium HumanMethylation450 BeadChips. *Genome Biol* 13:R44-2012-13-6-r44.

6. Chen YA, Lemire M, Choufani S, Butcher DT, Grafodatskaya D, Zanke BW, et al (2013): Discovery of cross-reactive probes and polymorphic CpGs in the Illumina Infinium HumanMethylation450 microarray. *Epigenetics* 8:203-209.

7. Chen EY, Tan CM, Kou Y, Duan Q, Wang Z, Meirelles GV, et al (2013): Enrichr: interactive and collaborative HTML5 gene list enrichment analysis tool. *BMC Bioinformatics* 14:128-2105-14-128.

8. van der Maaten, L. & Hinton, G. Visualizing Data using t-SNE. *Journal of Machine Learning Research* **9**, 2579-2605 (2008).
